# Drug Resistance Biomarkers and Their Clinical Applications in Childhood Acute Lymphoblastic Leukemia

**DOI:** 10.3389/fonc.2019.01496

**Published:** 2020-01-17

**Authors:** Narges Aberuyi, Soheila Rahgozar, Elaheh Sadat Ghodousi, Kamran Ghaedi

**Affiliations:** Division of Cellular and Molecular Biology, Department of Cell and Molecular Biology & Microbiology, Faculty of Biological Sciences and Technologies, University of Isfahan, Isfahan, Iran

**Keywords:** childhood acute lymphoblastic leukemia, multidrug resistance, prognostic biomarkers, predictive biomarkers, drug resistance biomarkers

## Abstract

Biomarkers are biological molecules found in body fluids or tissues, which can be considered as indications of a normal or abnormal process, or of a condition or disease. There are various types of biomarkers based on their application and molecular alterations. Treatment-sensitivity or drug resistance biomarkers include prognostic and predictive molecules with utmost importance in selecting appropriate treatment protocols and improving survival rates. Acute lymphoblastic leukemia (ALL) is the most prevalent hematological malignancy diagnosed in children with nearly 80% cure rate. Despite the favorable survival rates of childhood ALL (chALL), resistance to chemotherapeutic agents and, as a consequence, a dismal prognosis develops in a significant number of patients. Therefore, there are urgent needs to have robust, sensitive, and disease-specific molecular prognostic and predictive biomarkers, which could allow better risk classification and then better clinical results. In this article, we review the currently known drug resistance biomarkers, including somatic or germ line nucleic acids, epigenetic alterations, protein expressions and metabolic variations. Moreover, biomarkers with potential clinical applications are discussed.

## Introduction

**Figure 1:**
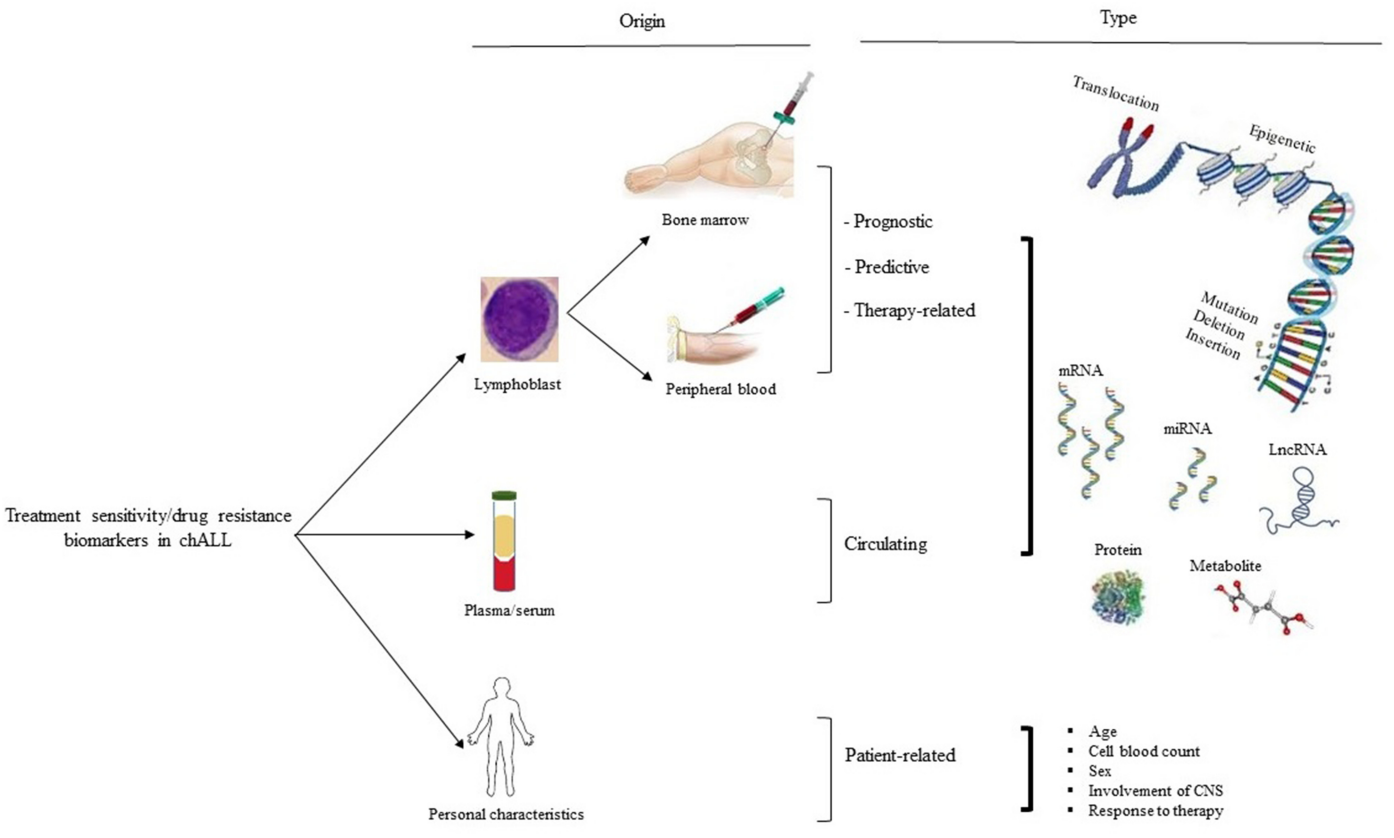
Treatment sensitivity/drug resistance biomarker in chALL. There are three types of treatment sensitivity/drug resistance biological markers based on their origins of detection, including patient lymphoblast, serum/plasma, and personal characteristics (such as age, cell blood count, sex, involvement of CNS, and response to therapy). Biomarkers related to lymphoblastic cells are subdivided into prognostic, predictive, and therapy-related biological markers. CNS, the central nervous system.

The National Institute of Health (NIH) defines biological markers or biomarkers as biological markers found in body fluids, such as blood or tissues, that are considered as signs of a normal or abnormal process, or of a condition or disease (1–3). Biomarkers typically differentiate patients from persons without the disease. They can be detected in the excretions or secretions including urine, stool, sputum, or nipple discharge, or in the circulation such as whole blood, plasma, or serum, and therefore easily, non-invasively and serially assessed. Biological markers can be tissue-derived and require either special imaging or biopsy for evaluation (Figure 1). They are assuming a growing role in all aspects of oncology including patient assessment, estimation of disease risk, screening for occult primary cancers, distinguishing malignant from benign tumors or one type of tumor from another, prediction and determination of the prognosis for patients who have been diagnosed with malignancy, and monitoring status of the disease either to detect recurrence or progression or determine response to treatment (1).

There are two major types of biomarkers: (1) Biomarkers of exposure which are used in cancer susceptibility and risk prediction, (2) Biomarkers of disease that are utilized in screening, diagnosis, and monitoring of disease progression (4). Cancer susceptibility and risk stratification biomarkers indicate those individuals at an increased risk of developing malignancy, who can be candidates for prevention investigations, such as: biomarkers of exposure to carcinogens, oxidative stress, inflammation and molecular alterations at DNA level (including some single nucleotide polymorphisms (SNPs) and mutations in specific genes) (5). There are many types of disease biomarkers based on their application, including: diagnostic, classification, staging, treatment sensitivity or drug resistance biomarkers, and biomarkers associated with disease aggressiveness and progression used in screening and monitoring of disease progression (3, 6, 7). Personalized medicine methods are being developed based on the cancer diagnostic and treatment sensitivity or resistance biomarkers at cellular levels. Using precise targets for diagnosis of cancers is of importance, since the current treatment approach is based on a targeted therapy (8, 9). Therefore, diagnostic and treatment sensitivity/drug resistance biomarkers are more important in clinical and cure rate of cancers.

Diagnostic biomarkers may be signals of the cancer presence and are recognized by characterizing molecular alterations and main mutations involved in cancer development and proliferation or any substance or process that is indicative of the presence of cancer in the body (2, 6). For example, changes in some protein expression levels can be used as the markers of early detection of B-ALL, including leucine-rich alpha-2-glycoprotein 1 (LRG1), thrombin (F2), alpha-2-macroglobulin (A2M), heparin cofactor II (SERPIND1), clusterin (CLU), alpha-1 antitrypsin (SERPINA1), complement C3 (C3), complement factor B (CFB), and alpha-2-antiplasmin (SERPINF2) (10). These biomarkers are useful in targeted therapies and in preventing toxicity related to standard (systemic) therapies. Detection of cancer at the early stages through diagnostic biomarkers significantly increases the chances of successful therapy.

Treatment sensitivity/drug resistance biomarkers can be considered as prognostic, predictive, therapy-related, circulating, and patient-related markers (Figure 1). Prognostic biomarkers assess outcome regardless of therapy type, whereas predictive markers estimate outcome of a specific treatment compared with another one. These biomarkers identify somatic or germ line nucleic acids (i.e., DNA, RNA, microRNA, or other non-coding RNAs such as long non-coding RNAs), epigenetic changes (i.e., DNA methylation), proteins (i.e., enzymes or receptors), antibody expressions, and even metabolic variations (1, 2, 6–8).

Acute lymphoblastic leukemia (ALL) is one of the most common hematologic cancers in children, responsible for 75% of all childhood leukemias (11–13). The contemporary treatment for the childhood ALL (chALL) is based on standard combination chemotherapy and particular preventive treatment of the central nervous system (CNS) (14). Despite the successful treatment rate of about 70-80%, the most difficult challenge in the treatment of chALL is still the risk of relapse in 20-30% of these children (15). The cure rate of chALL is related to early detection and stratification of individual patients into the risk groups of standard, low, intermediate, or high risks, based on the presence of special prognostic factors, genetic findings and response to treatment (14, 16).

Because of the lack of adequate initial symptoms, the clinical diagnosis of chALL requires a bone marrow biopsy/aspiration which is completely invasive, especially for children (17). Considering the laborious and painful nature of bone marrow biopsy/aspiration, reliable biomarkers for disease diagnosis and monitoring is appealing and of paramount importance (18). Appropriate biomarkers allow non-invasive monitoring of the patients and earlier diagnosis of disease, which may contribute to improved outcome. Furthermore, identifying new prognostic biomarkers is useful for the risk stratification of the patients and determining when to treat more aggressively or less intensively. Moreover, it may help developing novel drug targets and designing individualized therapies (5, 6). The current study mainly focuses on the prognostic biomarkers identified in chALL in order to help predicting response to treatment and adopting proper strategy for the disease management.

## ALL Prognostic Biomarkers

The development of multidrug resistance (MDR) phenomenon and relapsed ALL is the most common cause of therapy failure and the main cause of the increased morbidity and mortality in children with ALL (19, 20). According to the WHO classification of leukemic neoplasms, ALL patients are categorized into standard, low, intermediate, or high risk groups according to the identified molecular and/or cytogenetic markers (i.e., *MLL*-*AF4* and *BCR*-*ABL* rearrangements) and response to treatment (21). Leukemia minimal residual disease (mrd) level quantification is widely used for prediction of impending relapse and clinical outcomes, therapeutic hierarchy of chALL, and guiding clinicians to develop appropriate and efficient therapy options so that patients can avoid unnecessary chemical drug toxicity. Both quantitative polymerase chain reaction (QPCR) and flow cytometry analysis can be used to identify mrd. These techniques are sensitive, with the ability to detect one blast cell among 10^3^ to 10^6^ normal cells; robust; and reproducible. However, allele-specific QPCR is routinely used to detect mrd in chALL, using immunoglobulin heavy chain (IGH) or T-cell receptor (TCR) gene rearrangements (22, 23). Furthermore, the multiplex real time PCR (RT-PCR) is another useful, rapid and flexible molecular technique, which provides additional information for accurate diagnosis and prognosis of chALL, such as identifying translocations and mutations in *KRAS* gene and the acquired mutations in the *ABL1* kinase domain for predicting response to targeted treatments (8, 24). However, the number of identified fusion genes in acute leukemia is still limited.

RT-PCR assays show insufficient standardized cut-offs, and invasiveness of bone marrow aspiration which is painful for patient (25). Therefore, there is a huge interest in determining accurate disease-specific and sensitive biomarkers that are required for better risk assortment, predicting treatment response and distinguishing between indolent and aggressive disease (26). These biomarkers are essential for the assessment of the risk of relapse at diagnosis and could be useful in identification of patients requiring more intensive therapy (5, 16). The exact assignment of patients to various risk groups is critical to determine the premium therapeutic strategy for each patient and results in increased patient survival rate and reduced medical costs (27). Risk-based treatment is emphasized in therapeutic protocols for chALL to decrease the toxicity in low risk children and provide aggressive treatments for those with high risk of disease recurrence (21). Risk stratification adapted treatments using prognostic biomarkers will help to increase the cure rate (25). Remarkable advancement in molecular techniques and high throughput DNA sequencing has provided many nucleic acid-, epigenetic- and protein-based prognostic biomarkers which are described in below sections (9).

### Deoxyribonucleic Acid-Based Biomarkers

The fact that ALL develops only in a small number of individuals exposed to the specific environmental and lifestyle risk factors, indicates that the host genetic factors may have a key role in the genesis of leukemia (12, 28). Molecular modifications at the DNA level include numerical- and structural-chromosomal abnormalities such as rearrangements/translocations, point mutations/deletions or insertions, SNPs and gene replication (Table 1) (8). These genetic biomarkers can be somatic, recognized as mutations in DNA derived from tumor tissue, or germ line sequence variations, DNA isolated from whole blood, buccal cells, or sputum (1). Unlike protein markers, genetic biomarkers are more reproducible and less affected by intrinsic and extrinsic stimuli (6). Genomic alterations are a composite part of diagnosis and classification of hematological malignancies and have implications in the prognosis, risk stratification and selection of the appropriate therapy protocol based on the molecular changes (8). Currently, a very active area of tumor research is the use of genetic and epigenetic alterations in order to develop targeted therapies (58).

**Table 1:** Nucleic acid-based prognostic biomarkers at DNA and mRNA levels in chALL.

**DNA/mRNA**	**Type of biomarker/abnormality**	**Favorable (F)/Unfavorable (U) prognosis**	**Lineage**	**References**
**DNA**				
*iAMP21*	Amplification	U	B	(29)
*TCF3-PBX1(E2A-PBX1)*	Translocation	F	B	(30)
*BCR-ABL1, MLL-AF4, ETV6-RUNX1(TEL-AML1)*	Translocation	U	B	(31)
*MLL-AF9*(*MLLT3*), *MLL- MLLT1*			B, T	(32)
*IGH@- CEBP*	Translocation	U	B	(33)
*TLX1-TRD, TRB-TLX1*	Translocation	F	T	(34, 35)
*TLX3-CTIP2*	Translocation	Controversial	T	(35)
*LYL1-TCRB*	Translocation	U	T	(36)
*CALM-AF10*	Translocation	U	T	(37)
*ETV6, PAX5, CDKN2A/B*	Duplication/deletion	U	B	(38)
*CDKN2A*	Deletion	U	T	(39)
*CREBBP*	Mutation	U	B, T	(40)
*PTEN*	Mutation	U	T	(41)
*NOTCH1*	Mutation	F	T	(42)
*ERG*	Deletion	F	B	(29)
*JAK*	Mutation	U	B	(31)
*NRAS, KRAS, PTPN11, FLT3*	Mutation	U	B	(43)
*ABCC4*/*MRP4*	Polymorphism	U	B	(44)
*BAALC*	Polymorphism	U	B	(27)
*ABCB1/MDR1*	Polymorphism	U	B, T	(45–47)
*MRP1, MRP2, ABCG2/BCRP*	Polymorphism	U	B, T	(45)
*GATA3*	Polymorphism	U	B, T	(48, 49)
*Fas/CD95*/*APO*-1	Polymorphism	F	B, T	(50)
*GSTP1*	Polymorphism	U	B, T	(51)
*ARID5B*	Polymorphism	U	B	(52)
**mRNA**				
*ABCA2, ABCA3, ABCB1, ABCC1*,	Overexpression	U	B, T	(53)
*BAALC*	Overexpression	U	B, T	(15)
*HDAC3, HDAC7, HDAC9*	Overexpression	U	B, T	(54)
*Bmi-1*	Overexpression	U	B, T	(55)
*BIRC5, FOXM1, PAICS, TYMS, CAD, ATIC, GART*	Overexpression	U	B	(56)
*SALL4*	Overexpression	U	B	(57)

*iAMP21, Intrachromosomal amplification of chromosome 21; TCF3, Transcription factor 3; PBX1, Pre-B-cell leukemia homeobox 1; BCR, Breakpoint cluster region; ABL1, Abelson murine leukemia viral oncogene homolog 1; MLL, Myeloid/lymphoid leukemia or Mixed lineage leukemia; AF4, ALL1-fused gene from chromosome 4; ETV6, ETS Variant 6; RUNX1, Runt-related transcription factor 1; TEL, Translocation–ETS–leukemia; AML1, Acute myeloid leukemia 1; AF9, ALL1-fused gene from chromosome 9; MLLT3, myeloid/lymphoid or mixed-lineage leukemia (trithorax homolog, Drosophila); translocated to, 3; IGH@, Immunoglobulin heavy-chain locus; CEBP, CCAAT enhancer-binding protein; TLX1, T cell leukemia homeobox 1; TRD, T cell receptor delta locus; TRB, T cell receptor beta locus; TLX3, T cell leukemia homeobox 3; CTIP2, Coup-TF interacting protein 2; LYL1, Lymphoblastic leukemia derived sequence 1; TCRB, T-cell receptor beta chain; CALM; Calmodulin; AF10, ALL1-fused gene from chromosome; PAX5, Paired box gene 5;CDKN2A/B, Cyclin-dependent kinase Inhibitor 2A/B; IKZF1, IKAROS family zinc finger 1; PTEN, Phosphatase and tensin homolog; ERG, ETS transcription factor; JAK, Janus kinase 2; NRAS, Neuroblastoma rat sarcoma viral oncogene homolog; KRAS, Kirsten rat sarcoma viral oncogene homolog; PTPN11, Protein tyrosine phosphatase, non-receptor type 11; FLT3, Fms-like tyrosine kinase 3; CREBBP, CREB-binding protein; ABCC4/MRP4, ATP-binding cassette sub-family C member 4/Multidrug resistance-associated protein 4; BAALC, Brain and acute leukemia, cytoplasmic; ABCB1/MDR1, ATP-binding cassette sub-family B member 1 /Multidrug resistance protein 1;MRP1, Multidrug resistance-associated protein 1; MRP2, Multidrug resistance-associated protein 2; ABCG2/BCRP, ATP binding cassette sub-family G member 2/Breast cancer resistance protein; GATA3, GATA binding protein 3; APO-1, Accumulation of photosystem, protein 1;GSTP1, Glutathione S-transferase Pi 1; ARID5B:AT-rich interaction domain 5B; ABCA2, ATP binding cassette subfamily A member 2; ABCA3, ATP binding cassette subfamily A member 3; ABCB1, ATP binding cassette subfamily B member 1; ABCC1, ATP binding cassette subfamily C member 1; HDAC3, Histone deacetylase 3; HDAC7, Histone deacetylase 7; HDAC9, Histone deacetylase 9; Bmi-1, B cell-specific Moloney murine leukemia virus integration site 1; BIRC5, Baculoviral IAP repeat-containing protein 5; FOXM1, Forkhead box protein M1; PAICS, Phosphoribosyl-aminoimidazole carboxylase and phosphoribosyl-aminoimidazolesuccino-carboxamide synthase; TYMS, Thymidylate synthetase; CAD, Carbamoyl-phosphate synthetase 2, aspartate transcarbamylase, and dihydroorotase; ATIC, 5-aminoimidazole-4-carboxamide ribonucleotide formyl-transferase/IMP cyclohydrolase; GART, Phosphoribosyl-glycinamide formyl-transferase; SALL4, Spalt like transcription factor 4*.

Recurring genomic alterations have a crucial role to induce drug resistance at relapse (59). In fact, differences in clinical features and outcomes observed in chALL patients are thought to be the result of variety in the chromosomal abnormalities, genetic backgrounds, and mutations in leukemic blasts (45). In most therapeutic protocols, different genetic subgroups of chALL are treated following risk-adapted treatment, which is tailored to the children' relative high risk of recurrence (60). Recently, the number of genetic modifications found in ALL patients has been extremely increased by developed multi next-generation sequencing (NGS) methods such as whole-genome sequencing and transcriptome sequencing (31). Several numerical chromosome changes have been recognized in chALL, including hypodiploidy, hyperdiploidy, near-haploidy, and complex karyotypes (61). The prevalence of hyperdiploidy is more than near-haploidy, hypodiploidy, and complex karyotypes in pediatric B cell precursor (BCP)-ALL and it is associated with good treatment response (29, 33, 61). Intrachromosomal amplification is another chromosome abnormality related to the treatment response. For example, intrachromosomal amplification of chromosome 21 (iAMP21) is related to unfavorable prognosis in childhood BCP-ALL (29). iAMP21 is defined as amplification of the 5.1-Mb common region containing genes mapping to the Down Syndrome Critical Region (DSCR), RUNX1 and miR-802. Childhood BCP-ALL with iAMP21 consistently has 3 or more copies of the *RUNX1* gene (62). Deletion of *IKZF1, CDKN2A, RB1*, and *PAX5* genes are considered as other genetic alterations related to iAMP21 (30). Translocations, mutations and polymorphisms are the most common DNA level prognostic biomarkers in chALL.

### Translocations/Rearrangements

Chromosomal irregularities mostly include non-random chromosomal translocations, which may generate novel fusion genes or cause inopportune gene expression of proto-oncogenes or altered proteins (21). Some of the common genetic events, such as translocations, are used for risk stratification and therapy assignment in chALL. Chromosomal translocations, such as *BCR*-*ABL1, ETV6*-*RUNX1*, and *E2A*-*PBX1*, occur in about 80% of childhood B-ALL and can be detected using interphase fluorescence *in situ* hybridization (FISH) and routine cytogenetic analysis (31). However, ~50% of T-ALL children have normal karyotype and cytogenetic (63). Here, we discuss some frequent chromosomal translocations which are known as prognostic biomarkers in chALL.

E2A, also known as TCF3, is located at chromosome 19p13.3. This basic helix-loop-helix transcription factor is essential for development and differentiation of B-cell. The chromosomal translocation t(1;19)(q23;p13) is the most prevalent translocation in *E2A* gene and appears in ~5–7% of childhood B-ALL cases. The resulting E2A-PBX1 is a chimeric fusion protein created by the amino-terminal transactivation domains of E2A and the DNA-binding homeodomain of PBX1 (30). Another *E2A* related translocation, t(17;19)(q22;p13), happens rarely among childhood B-ALL and includes the amino-terminal transactivation domains of E2A and the leucine zipper dimerization domain of HLF (31, 64). In pediatric B-ALL, t(1;19) is related to a favorable outcome, while chromosomal translocation t(17;19) is associated with a poor prognosis (30, 65).

The reciprocal translocation t(9;22)(q34;q11) results in the production of a Philadelphia chromosome and the expression of tyrosine kinase BCR-ABL1 chimeric fusion protein, the *ABL1* oncogene on chromosome 9 and the *BCR* gene on chromosome 22. This translocation rarely occurs in children and the fusion protein has ABL1 kinase activity and localizes in the cell nucleus. Depending on the breakpoint site of the *BCR* gene, the BCR-ABL chimeric proteins may possess various molecular weights. A shorter form, p190, is observed in childhood Ph+ B-ALL and is associated with a worse outcome and a 5-year overall survival (OS) of <10% (29, 31, 66).

The most frequent chromosomal translocation involving the mixed lineage leukemia (*MLL*) gene in B-ALL is t(4;11)(q21;q23), which is correlated with very poor prognosis for infants under 1 year of age, a great number of whom experience relapse. However, this translocation is associated with more favorable prognosis for children between the ages of 1 and 9 years or those of 10 years of age or older, and results in an *MLL-AF4 (AFF1)* fusion gene. Additionally, *MLL-AF4* consists of more than 50% of the translocations and is more frequent (~24%) in children who have received chemotherapy for other tumors. Generally, this translocation is associated with an increased risk of recurrence in childhood B-ALL (29, 31, 67). Other 11q23 translocations in leukemia are t(9;11)(p22;q23) and t(11;19)(q23;p13.3), which create *MLL-AF9*(*MLLT3*) and *MLL- MLLT1* fusion genes, respectively. In T-ALL and non-infant B-ALL patients, t(11;19)(q23;p13.3) translocation is correlated with favorable outcome, while in infant B-ALL cases, this genetic abnormality is associated with poor outcome (32).

*ETV6*, formerly known as *TEL*, is located at chromosome 12p13 and encodes an ETS family transcriptional repressor protein. It is commonly fused with other genes in human lymphoid or myeloid leukemia (68). *RUNX1*, previously known as *AML1*, located at chromosome 21q22, encodes a transcription factor protein that is important for embryonic hematopoiesis as well as B-cell differentiation in adult blood cell progenitors. Chromosomal translocation t(12;21)(p13;q22) leads to the ETV6-RUNX1 fusion protein, consisting of the N-terminal non-DNA-binding domain of ETV6 combined with RUNX1, and is the most common alteration in childhood B-ALL (~30%), but it is rarely observed in T-ALL (69). Children with B-ALL correlated with ETV6-RUNX1 show a highly good response to treatment (31). Despite the favorable prognostic factor of *ETV6*-*RUNX1* translocation, relapse occurs in up to 20% of these subtypes demonstrating this cytogenic abnormality (70). Furthermore, recurrent translocations of the immunoglobulin heavy-chain locus (*IGH@)* are relatively rare but have been well-documented in B-ALL. Translocations of *IGH@* with each of the five members of the *CEBP* transcription factor family have been identified in childhood B-ALL. However, the *CEBP*/*IGH* fusion, as a result of t(8;14)(q11;q32), is the most frequent translocation reported in pediatric B-ALL. This chromosomal alteration occurs either as a single acquired abnormality, or in conjunction with Down syndrome or t(9;22)(q34;q11). Chromosomal translocation t(8;14)(q24;q32) is correlated with a very inferior outcome and relapse (33).

TLX1 (also known as HOX11), located at chromosome 10q24.31, is a HOX transcription factor, which plays essential role in cell fate and differentiation during normal development. Genetic rearrangement t(10;14)(q24;q11.2) occurs in ~10% of childhood T-ALLs and induces dysregulation of *TLX1* expression in these patients (34). Another translocation with aberrant expression of HOX11 is t(7;10)(q35;q24) (35). It has been demonstrated that T-ALL children with HOX11 expression show better survival (35, 71).

Another more frequent translocation in pediatric T-ALL is t(5;14)(q35;q32), observed in ~>20% of these patients. However, this genetic alteration encodes HOX11L2-BCL11B fusion protein (also known as TLX3-CTIP2) and leads to HOX11L2 activation, which is a homeobox transcription factor (72). The prognostic significance of this rearrangement remains controversial. There is an ongoing debate about the prognostic impact of t(5;14)(q35;q32) in T-ALL. Both, positive (35) and negative (73, 74) correlations, even no association (75) between the *HOX11L2* expression and T-ALL prognosis are published recently. These controversial data may be due to the sample size of the cohort investigated (35).

Lymphoblastic leukemia-derived sequence 1 (*LYL1*) gene is mapped at chromosome 19p13 and a basic helix-loop-helix (bHLH) transcription factor in T-ALL. The chromosomal translocation t(7;19)(q35; p13) leads to truncation of the *LYL1* gene and creates *LYL1-TCRB* fusion gene. This translocation is associated with poor prognosis in pediatric T-ALL (36). Furthermore, another rare frequent translocation correlated with poor prognosis in pediatric T-ALL is t(10;11)(p13;q14), which creates *CALM-AF10* fusion gene (37).

Admittedly, the genetic basis of the disease still remains unknown in a significant fraction of pediatric BCP-ALL and the response to treatment is unpredictable at the time of diagnosis in these patients (29). Therefore, finding of new prognostic biomarkers is critical for better risk assortment and improving the clinical outcome in chALL.

### Other Genetic Mutations

Although chromosomal rearrangements are used as predictive factors of chALL, recurrent genetic alterations are associated with ALL response to treatment. The nature of these mutations includes changes of single base pairs to small deletions/insertions, duplications and copy number variation of DNA sequences. Minor deletions and amplifications are more precisely identified using genome wide DNA copy number profiling studies. Until now, more than fifty different recurring genetic abnormalities have been recognized. The most frequently affected genes are involved in lymphocyte differentiation (*PAX5, IKZF1*, and *EBF1*), lymphoid signaling (*BTLA, CD200, TOX, BLNK, VPREB1*), transcriptional regulation (*ETV6, ERG*), regulation of cell-cycle and tumor suppression (*CDKN2A*/*CDKN2B, RB1, PTEN*), and drug responsiveness (the glucocorticoid (GC) receptor *NR3C1*) (76). These alterations can be also determined by PCR. In this part, the most frequent mutations observed in the prognostic biomarkers of chALL will be reviewed.

Recently, our group demonstrated that BCP-ALL children with at least one of the genetic alterations including *ETV6* (12p13.2) duplication, *PAX5* (9p13.2) deletion, and *CDKN2A/B* (9p21.3) deletion must be classified as those with high-risk ALL, which have worse 3-year disease free survival (3-DFS) (38). PAX5 alteration, a B-lineage specific transcription factor, is one of the most common genetic aberrations in B-ALL (~30%). There are various genetic alterations correlated with *PAX5* gene, including point mutations, monoallelic deletions, and translocations. *PAX5* deletions are more common and correlated with *E2A-PBX1, BCR-ABL1* and complex karyotype with secondary genetic changes; while *PAX5* rearrangements are relatively rare (2.5% of B-ALL cases). *CDKN2A* (*MTS1*) and *CDKN2B* (*MTS2*) are well-known tumor suppressor genes that encode p16^INK4a^/p14^ARF^ and p15^INK4b^ proteins, respectively. Deletion of the 9p21 chromosomal region leads to deletion in these proteins, controlling G1/S cell-cycle progression, and are identified in 21–36% of pediatric B-ALL (consisting of 42 children and 60 adults) (31). Furthermore, *CDKN2A* deletion is also correlated with poor event-free survival (EFS) in pediatric T-ALL (39).

CREBBP is a transcriptional activator with various functions such as regulation of protein turnover and acetylation of histone and non-histone targets through its E4 polyubiquitin ligase and acetyl transferase activities, respectively. The activated transcription of gene *CREBBP* is associated with poor response to treatment in chALL (including 82 T-ALL and 88 B-ALL) (40). Besides, deletions in *MTA1*/14q32.33 and 15q13.2 as well as gain on 1p36.11 are correlated with shorter OS in chALL (including 115 B-ALL and 27 T-ALL) (77).

*NOTCH1* gene, located at chromosome 9, encodes a single-pass heterodimeric transmembrane receptor with essential role in T-cell development. Chromosomal translocation t(7;9)(q34;q34.3) involves *NOTCH1* gene in a limited number of T-ALL patients. However, activating mutations of *NOTCH1* occur in up to 50% of pediatric T-ALL and involve either the heterodimer domain (HD) or the PEST domain of this protein, leading to ligand independent activation of NOTCH1. Interestingly, *NOTCH1* activating mutations are associated with a favorable mrd and long-term outcome as well as prednisone response in these children (42).

The majority of *PTEN* tumor suppressor gene alterations in T-ALL are mutations resulting in truncated membrane binding C2-domain or deletions affecting the entire *PTEN* locus. A study has shown that small deletions result in inactivated *PTEN* by affecting only a few exons in about 8% of childhood T-ALL. The *PTEN* alterations (major deletions or mutations) are correlated with poor prognosis, leading to hyperactive PI3K–AKT signaling that impairs apoptosis and drives increased cell proliferation and metabolism in T-ALL patients (41). It has been suggested that *PTEN* deletion has more adverse outcome than mutations which preserve the N-terminal phosphatase domain (78).

Somatic genetic alterations of *IKZF1*, located at 7p13-p11.1 and encoding the lymphoid transcription factor IKAROS, are the most prevalent genetic lesions in high-risk BCP-ALL and are considered as very frequent events in *BCR*-*ABL1*–positive ALL (75 to 90%). Rare and frequent *IKZF1* deletions, affecting exons 4-7 and exons 1-8, are correlated with poor prognosis, high risk of recurrence and EFS rates of below 50% (79–81). *IKZF1* mutations/deletions lead to loss of IKZF1 function and drug resistance by affecting expression of the various molecules in different signaling pathways including cell adhesion (*RHO, CTNND1, FAK, CD90(THY1)*, and *ITGA5*), metabolic pathways (*GLUT1/3/6, INSR, HK2, AMPK, HK3, LKB1, G6PD, TXNIP, NR3C1*, and *CNR2*), GR target gene regulation (*NR3C1, EMP1, EMP1*, and *IK6*), and PI3K/AKT/mTOR pathway (*PIK3CD* and *PIK3C2B*) (82). Overexpression of cell adhesion molecules, creating aberrant cell-cell and cell-stroma interactions and acquisition of stem cell-like properties, mediates extravascular invasion, residence in the niche, activation of integrin signaling pathways, and reduced sensitivity to tyrosine kinase inhibitors. Overexpression and binding of THY1 at the cell surface lead to Src and FAK activation. FAK activation results in migration, proliferation and survival, due to the activation of various signaling pathways such as Rac/Rho and PI3K/AKT. Furthermore, IKZF1-related dysregulation of *NR3C1* gene, encoding GC receptor, involves in GC resistance (83). However, coding *IKZF1* mutations have been identified in familial B-ALL and ~0.9% of presumed sporadic pediatric B-ALL, most variants of which adversely affect IKZF1 function and response to treatment. Detection of *IKZF1* alterations at diagnosis may be considered as a negative prognostic factor and it can be useful in identifying B-ALL children with high risk of treatment failure (79, 80).

Recent genomic profiling studies of chALL have identified a recurrent V(D)J-mediated intragenic deletion of the *ERG* gene (*ERG*^*del*^) in about 7% of pediatric BCP-ALL, coding a member of the *ETS* transcription factor family with a main role in hematopoiesis (84). It has been observed that *ERG*^*del*^ is associated with older age, aberrant CD2 expression and frequent *IKZF1*Δ4-7 deletions. In one research, *ERG*^*del*^ chALL showed a favorable outcome, with an 8-year EFS and 8-year OS of 86.4 and 95.6%, respectively. In addition, presence of *ERG*^*del*^ in BCP-ALL children with *IKZF1* Δ4-7 deletions showed a better disease outcome. Therefore, *ERG*^*del*^ can be used as a positive prognostic biomarker for patients with *IKZF1* status (29).

Mutations of Janus kinases (JAKs), protein tyrosine kinases and key players in the JAK-STAT pathway, were initially identified in B-ALL correlated with Down syndrome. However, about 10% of B-ALLs without Down syndrome is also have the heterozygous somatic mutations of *JAKs*. These alterations happen in highly conserved residues in the kinase and pseudo-kinase domain, resulting in constitutive kinase activation, and are highly correlated with aberrant expression of the cytokine receptors in B-ALL patients. It has been shown that *JAK* mutation is correlated with relapse in B-ALL and ~70% of children with *JAK* mutation carry concomitant deletion of *CDKN2A/B* and/or *IKZF1* (31).

RAS pathway alterations have been associated with chemotherapy resistance and inferior outcome in BCP-ALL and the large majority of aberrations (98%) occur in *NRAS, KRAS, PTPN11*, and *FLT3* genes. High hyperdiploid, *MLL*-*AF4*-rearranged, *BCR*-*ABL1*-like and B-other cases have high alteration frequencies, while *ETV6*-*RUNX1* cases have moderate frequencies in RAS pathway. Furthermore, researchers found that *TCF3*-*PBX1* and *BCR*-*ABL1*-rearranged cases show rare frequencies in this pathway. RAS pathway mutations are related to unfavorable outcome and can be used as prognostic biomarkers in pediatric BCP-ALL (43).

### Polymorphisms

Polymorphisms affect clinical outcome in chALL patients and are considered as potential prognostic biomarkers in this regard (45). Different genetic polymorphisms have been recognized as probable risk factors for hematological malignancies. In one study, 102 mrd–associated SNPs are detected in chALL using a genome-wide interrogation (85). Genetic polymorphisms related to genes mediating transportation and metabolism of drug have been indicated to be of importance in the difference in survival after cancer treatment (45). Some of the new polymorphism biomarkers are given below.

In recent years, the main focus of our studies has been placed on chALL and the cellular and molecular factors contributing to ALL MDR. We have identified SNP rs2274407 (G912T; K304N) located in the 3′ splice acceptor site of exon 8 of ATP-binding cassette subfamily C member 4 (*ABCC4* or *MRP4*) pre-mRNA and *ABCC4 G*912T allele carriers (G/T and T/T genotypes), which are correlated with worse 3-DFS in pediatric BCP-ALL. Additionally, it is identified that the T allele of rs2274407 may have functional impact on the aberrant splicing of *ABCC4* mRNA. Bioinformatics analyses and *in vitro* studies suggested that the G912T allele can result in the elimination of about 250–300 bp in exon 8 and reduction of *ABCC4* expression in lymphoblastic cells (44). Some other polymorphisms including G2269A, C912A, G559T, T1393C, and A934C have been identified additionally in the *ABCC4* gene, which may be associated with outcomes in chALL (including 94 chALL containing B- and T-ALL with unidentified percentage) (86, 87).

Our group has also indicated that the rs62527607 [GT] SNP of Brain and Acute Leukemia, Cytoplasmic (*BAALC*) gene can be used as a novel negative prognostic biomarker in childhood ALL. The GT genotype may lead to an increase in the risk of drug resistance in pre-B-ALL and T-ALL cases by 2.86 and 8.75 folds, respectively, compared with those with GG genotype. However, patients who have GG genotype show no difference in response to chemotherapy (27).

Evaluating gene polymorphisms in ABC transporters can help predict the outcome of ALL and optimize the efficacy of chemotherapy in ALL patients. In one study, the SNPs related to the genes of *MDR1*/*ABCB1, MRP1, MRP2*, and *BCRP/ABCG2* were investigated in 82 chALL patients (including 6 T-ALL and 76 B-ALL). It has been demonstrated that a combination of *MDR1* C1236T, *MRP2* G1249A, and/or *BCRP* G34A SNPs is remarkably correlated with decreased EFS and OS (45). Some other studies have also shown the correlation between polymorphisms of *MDR1* and risk of relapse in chALL (including 522 and 207 chALL containing B- and T-ALL with unidentified percentage). The risk of relapse is increased for patients with the 1199GA variant vs. 1199GG, the 3435CC variants vs. 3435CT/TT and the 1236CC variant vs. 1236CT/TT (46, 47). *MDR1* gene is a member of the ATP-binding cassette (ABC) family encoding P-glycoprotein (P-gp), which plays an important role in MDR of cancer cells by promoting cellular efflux of structurally and functionally different chemotherapeutic regimens, such as anthracyclines, taxanes, and vinca alkaloids (88). Some genetic polymorphisms in *MDR1* affect the intracellular expression levels of the P-gp protein, thereby resulting in MDR of pediatric ALL. Some *MDR1* gene polymorphisms, such as C3435T, observed in exon 26, have been revealed to change the normal conformation of the P-gp protein, which inhibits its substrate-binding properties contributing to worse clinical outcome. Additionally, a number of *MDR1* gene polymorphisms, including C3435T and G2677G/A, have been suggested to be correlated with the pharmacokinetics of the P-gp substrates including cyclosporine, digoxin, and fexofenadine. This study suggests a regulatory role for these SNPs in drug metabolism and ALL related SNPs (45).

The possible role of *GATA3* rs3824662 polymorphism as a prognostic biomarker has been investigated in chALL (including 98 B-ALL and 18 T-ALL). *GATA3* polymorphism which is located around the tissue-specific enhancer, is remarkably associated with the expression level of GATA3. The AA genotype is associated with significantly higher *GATA3* mRNA levels and with shorter DFS, which increases incidence of relapse and poor prognosis in chALL. The regulatory network of *GATA3* has also been shown in T- and B-ALL (48, 49).

GG genotype at −670 A/G position of *Fas* gene promoter is correlated with liver involvement in ALL patients (including 142 chALL containing B- and T-ALL with unidentified percentage). Fas or CD95/APO-1, a member of the superfamily of tumor necrosis factor receptors, is one of the significant molecules contributing to the extrinsic pathway of apoptosis (50). Binding apoptotic ligands to Fas receptor leads to homo-trimerization of this receptor, signaling transduction, death complex formation, and cell death (89). It is suggested that A to G substitution at the mentioned position leads to *Fas* dysregulation and resistance to treatment. As a result, *Fas* GG genotype at this position can be considered as a good prognostic biomarker in children with ALL (50).

Leonardi et al., studied the predictive value of germline polymorphisms in chALL (including 140 chALL containing B- and T-ALL with unidentified percentage). Glutathione S-transferases (GSTs) are enzymes which play role in detoxification. It was proved that patients with a glutathione S-transferase P1 (*GSTP1*) c.313A>G genotype have an increased risk of relapse, and a shortened Recurrence-Free Survival (RFS) was observed in homozygote G patients. The association was even stronger when the combined genotypes of *GSTP1* c.313GG, *GSTT1* null and *GSTM1* null was considered. In other words, patients who have 2/3 risk-allele genotypes are more prone to the risk of relapse and worse RFS. In fact, *GSTP1* c.313A >G SNP modifies the enzyme activity, and *GSTT1* and *GSTM1* genes with a deletion polymorphism result in no activity of the enzyme. These prognostic biomarkers could improve risk stratification and reduce the number of relapses and deaths in chALL (51). Furthermore, eight *ARID5B* SNPs are associated with relapse and worse treatment outcome in pediatric BCP-ALL, including rs6479778, rs2893881, rs4948488, rs2393782, rs6479779, rs7923074, rs10821938, and rs17215180 with T, C, C, G, C, T, A and C risk alleles, respectively. The first six SNPs are associated with lower OS and can be considered as prognostic factors in pediatric BCP-ALL (52).

### Ribonucleic Acid-Based Biomarkers

Changes at RNA level, occurs during RNA transcription or gene expression (8). Gene expression studies in chALL and the analysis of the DNA level alterations, have emphasized the great heterogeneity of this malignancy and made the possibility for differential diagnosis and identifying new sub-types of leukemia for better risk stratification (76). Various high-throughput methods are used to assess differential gene expression at RNA level in leukemia. Two of the most common methods include microarrays and next generation sequencing (90). Since high-throughput technologies remain prone to systematic errors, their clinical applications are limited and need to be confirmed using alternate methods, which are mostly included northern blot analysis and real time PCR (91, 92). Here, some of the gene expression alterations, known as prognostic biomarkers in chALL are reviewed. The related genes include: *ABCA2, ABCA3, ABCB1, ABCC1, BAALC, Survivin* (*BIRC5*), *FOXM1, PAICS, TYMS, CAD, ATIC, GART, HDACs, Bmi-1*, and Spalt-like transcription factor 4 (*SALL4*) (Table 1).

Possible association of the patient response to therapy with the mRNA expression levels of several ABC transporters including *ABCA2, ABCA3, ABCB1*/*MDR1, MRP1*/*ABCC1, MRP3*/*ABCC3, ABCG2*/*BCRP*, and *MVP*/*LRP*, has been previously investigated in chALL (including 21 B-ALL and 6 T-ALL). A significant positive correlation was observed between mRNA expression profiles of *ABCA2, ABCA3, MDR1*, and *MRP1* genes and positive mrd measured a year after treatment. Based on the statistical analyses and performed RT-PCR assays, it was shown that the overexpression of these genes may result in an increased risk of positive mrd by 15-, 6.25-, 12,-and 9-fold, respectively, suggesting a poor prognostic impact (53). Furthermore, *BAALC* has been introduced as a novel poor prognostic molecular marker in chALL (consisting of 23 B-ALL and 5 T-ALL). Our results demonstrated that the high expression level of *BAALC* is also associated with the positive mrd measured a year after treatment. Moreover, a positive association was determined between mRNA expression levels of *BAALC* and *MDR1*/*ABCB1* (15).

Higher expression levels of some histone deacetylases (*HDACs*) have been detected in chALL including *HDAC2, HDAC3, HDAC8, HDAC6*, and *HDAC7*. Overexpression of *HDAC1* and *HDAC4* have been shown in T-ALL, while *HDAC5* has higher expression in B-lineage ALL. As well as that, *HDAC3* overexpression is associated with a significant lower 5-year EFS in T-ALL patients (54). Increased expression level of *HDAC4* is correlated with high initial leukocyte count, T-ALL phenotype and prednisone poor-response in chALL (containing 73 B-ALL and 19 T-ALL) (93). Additionally, high expression levels of two other histone deacetylases, *HDAC7* and *HADC9*, are associated with a decreased 5-year EFS in B-lineage CD10-positive patients. Thus, *HDAC7* and *HADC9* overexpression is correlated with poor prognosis in chALL (including 78 B-ALL and 16 T-ALL). Therefore, they can be prognostic factors and promising therapeutic targets for the treatment of chALL (54).

It has been recently identified that ALL patients with up-regulated proto-oncogene *Bmi-1* at the time of diagnosis, show lower RFS rate (75.8%) than patients with down-regulated expression level of *Bmi-1* (94.1%). Furthermore, there is a significant positive association between increased level of *Bmi-1* and poor response to prednisone in chALL (consisting of 66 B-ALL and 11 T-ALL). The B cell-specific moloney murine leukemia virus insertion site 1 or Bmi-1 is a member of the Polycomb-group (PcG) family, which is known to be an oncogene and its expression can be a novel prognostic biomarker in children with ALL (55).

Some differential gene expressions are associated with relapse or its timing. As up-regulation of *BIRC5* (an apoptosis inhibitor/cell-cycle regulator) and *FOXM1* (an oncogenic transcription factor) genes were identified in relapsed BCP-ALL, these genes can be introduced as biomarkers/indicators of poor prognosis. Up-regulation of some genes involved in folate metabolism and nucleotide biosynthesis, including *PAICS, TYMS, CAD, ATIC*, and *GART*, has been observed in late relapse (56).

Up-regulation of SALL4 protein due to its CpG island hypomethylation, was reported in pediatric BCP-ALL. SALL4 is a newly identified zinc-finger transcriptional factor which is essential for embryonic development. This protein plays a key role in the B-ALL cell survival and can be considered as a potential novel target in the treatment of B-ALL (57). Furthermore, some studies reported that SALL4 can regulate *Bmi-1*, as the mRNA expression of *Bmi-1* is positively associated with that of *SALL4* (55).

### miRNA Levels Alteration

MicroRNAs, recently discovered non-coding endogenous single-stranded RNAs (composed of 19-24 nucleotides), show dysregulation during hematopoiesis and thereby have role in leukemogenesis (14, 94). These micro markers have proven to be very promising chALL biomarkers and have widespread clinical applications because of their stability and resistance to degradation in not only blood, but also various biological specimens (such as tissue, sputum, stool and formalin-fixed paraffin-embedded (FFPE) specimens), easily measured, and most importantly, their quantities correlates with the presence of the malignancy or with clinically relevant malignancy features (8, 14, 95–97). Recent studies have revealed that extracellular circulating miRNA levels in secreted membrane vesicles (microvesicles and exosomes), blood serum, and other body fluids can be used as biomarkers to diagnose, classify and predict prognosis of chALL (96, 98–101). miRNA expression dysregulation in cancers is because of different mechanisms, such as deletion or amplification of miRNA related genes, abnormal transcriptional control of miRNAs, epigenetic dysregulation and defects in the biogenesis machinery of miRNA (102). Some miRNAs might be up-regulated in chALL, and thus are suggested to have an oncogenic potential. On the other hand, some of them might be down-regulated in these patients, and are proposed to be tumor suppressors. Some examples of oncogenic miRNAs in chALL are miR-203 and miR-125b. Some tumor suppressor miRNAs are miR-181a, miR-326, and miR-200c, which show frequently down-regulation in chALL (97, 98). MicroRNA profiling can also be correlated with relevant clinical information, such as disease aggressiveness and metastatic potential, patient response to therapy, time-to-relapse and OS, and other clinical markers in chALL (101). Based on some investigations, not only can miRNAs regulate and sensitize the MDR phenotype, but also can be served as prognostic markers with potential implications in therapeutic interventions as drugs or therapeutic targets in chALL (103). Moreover, the identification of some prognostic miRNAs in patients can be used to predict prognosis and monitor cancer recurrence during their follow-up (14). Some new technologies are developed for the high-throughput identification of miRNAs in the diagnosis and prognosis of cancer, such as power-free microfluidic chip, electrically magnetic-controllable electrochemical biosensors, bead-based suspension array and one-step RT-PCR (8). A growing number of publications has revealed many examples of different miRNA profiles that provide potential prognostic and diagnostic biomarkers in chALL. As it is not plausible for this paper to review all of these studies, some of the critical miRNAs are introduced as follows. More investigated miRNAs are listed in Table 2.

**Table 2:** Nucleic acid-based prognostic biomarkers at miRNA and lncRNA levels in chALL.

**miRNA/lncRNA**	**Type of biomarker/abnormality**	**Favorable (F)/** **Unfavorable (U) prognosis**	**Lineage**	**References**
**miRNAs**				
*miR-125b1*	Upregulation	U	B	(31)
*miR-100, miR-99a*	Downregulation	U	T*, MLL*-rearrangement, *BCR-ABL*	(104)
*miR-335*	Downregulation	U	B, T	(105)
*miR-326*	Downregulation	U	B, T	(97)
*miR-335-3p*	Downregulation	U	B, T	(106)
*miR-7, miR-216, miR100*	Upregulation	U	B, T	(107)
*miR-486, miR-191, miR-150, miR-487, miR-342*	Downregulation	U	B, T	(107)
*miR-128b*	Upregulation	F	B, T	(108)
*miR-223*	Downregulation	U	B, T	(108)
*miR-24*	Downregulation	U	B, T	(109)
*miR-16*	Downregulation	F	B, T	(110)
*miR-33, miR-215, miR-369-5p, miR496, miR-518, miR-599*	Upregulation	U	B, T	(111)
*miR-10a, miR-134, miR-214, miR-484, miR-572, miR-580, miR-624, miR-627*	Upregulation	F	B, T	(111)
*miR-210*	Downregulation	U	B, T	(112)
*miR143, miR-182*	Downregulation	U	B, T	(113)
*miR-155a*	Upregulation	U	B, T	(114)
**LncRNAs**				
*NEAT1* and *MALAT1*	Upregulation	U	B, T	(106)
*BALR-6, LINC0098*	Upregulation	F	B	(115)
*RP11-137H2.4, AC156455.1*., *KB-208E9.1, CTA-331P3.1*	Upregulation	U	B	(116)
*RP11- 624C23.1, RP11-203E8, RP11-446E9*	Downregulation	U	B	(117)

*NEAT1, Nuclear paraspeckle assembly transcript 1; MALAT1, Metastasis associated lung adenocarcinoma transcript 1*.

*MiRNA-125b1*, also known as *miR-125-1*, is mapped at chromosome 11q24.1, which is the first miRNA identified in B-ALL and inserted into the rearranged IgH in some B-ALL patients. This translocation leads to the overexpression of *miR-125b*, which may play role as a negative regulator of *p53*, and can be considered as a poor prognostic micro marker for clinical outcome prediction (31).

Downregulation of *miR-100* and *miR-994* were found in pediatric T-ALL and children with the *MLL*-rearrangement and *BCR-ABL* fusion genes. It has been revealed that dysregulation of these miRNAs was also correlated with worse survival and prognosis in these children. Both miRNAs act as tumor suppressor genes and target *FKBP51*, involving in cell proliferation and apoptosis by impairing GC receptor translocation and reducing GC receptor activity and signaling pathways (104).

*MiRNA-335* downregulation is correlated with worse 5-year EFS and GC resistance in chALL (including 56 B-ALL and 7 T-ALL). Since *miR-335* targets *MAPK1*, its downregulation results in the upregulation of *MAPK1*, MEK/ERK map kinase pathway-mediated survival, proliferation and drugresistance of lymphoblast cells (105).

Our group has identified that miR-326 was downregulated in chALLs with MDR phenotype (36 B-ALL and 4 T-ALL). Furthermore, we demonstrated downregulation of miR-335-3p in the chALL MDR patients (58 B-ALL and 6 T-ALL). Interestingly, our data revealed that, there is a possibly causative correlation between the downregulation of miR-326 and miR-335-3p, increased risk of drug resistance and ALL relapse, and upregulation of ABCA2 and ABCA3 transporters, respectively (97, 106).

### Long Non-coding RNAs

Long non-coding RNAs, also known as lncRNAs (non-coding RNAs longer than 200nt) are emerging key regulators of numerous cellular processes such as gene expression (118). Different studies have already shown that changes in the expression of lncRNAs contribute to malignancies' development and progression, and can be utilized as diagnostic and prognostic markers in many cancers including ALL (Table 2) (119). Since the expression of lncRNAs is more tissue and cell type specific than that of protein coding mRNAs (120), these transcripts are potentially highly specific diagnostic/prognostic biological markers for used for the classification of cancer subtypes and stratify patients for clinical trials and therapeutic protocols (121).

A recent study by our group has shown that *NEAT1* and *MALAT1* lncRNAs *miR-335-3p* can be used as prognostic biomarkers in chALL (58 B-ALL and 6 T-ALL) (106). The expression of other four lncRNAs *BALR-1, LINC0098, BALR-6*, and *BALR-2* correlates with cytogenetic abnormalities, disease subgroups, OS and response to treatment in pediatric BCP-ALL. In addition, *BALR-6* and *LINC0098* up-regulation have been identified in patients with t(12;21) (115).

Another study reported up-regulation of five lncRNAs in BCP-ALL, including *RP11-68I18.10, AC156455.1, RP11-137H2.4, CTA-331P3.1*, and *KB-208E9.1*. These lncRNAs have significant effect on hallmark traits of cancer, such as migration, treatment response, apoptosis, and cell proliferation. The two lncRNAs, *RP11-137H2.4* and *RP11-68I18.10*, are correlated with proliferation, while *RP11-137H2.4, AC156455.1, KB-208E9.1*, or *CTA-331P3.1* are associated with apoptosis and drug resistance. In particular, knockout of lncRNA *RP11-137H2.4* effectively involves in inhibition of cell proliferation and migration and sensitive GC-resistant BCP-ALL cells to GC. This lncRNA has prognostic role and can be critical for the development of novel therapies to put an end to GC resistance in childhood BCP-ALL (116).

In BCP-ALL, three lncRNAs were reported to be down-regulated, namely *RP11-624C23.1, RP11-446E9*, and *RP11-203E8*. Restoring the expression of all these lncRNAs in leukemic cell lines has dramatic effect on cancer hallmark cellular phenotypes. Interestingly, *RP11-203E8* and *RP11-624C23.1* have a very similar effect on DNA damage response. Cells with up-regulation of *RP11-624C23.1* or *RP11-203E8* specifically display decreased γ-H2A.X and increased apoptosis levels than control cells in response to genotoxic stress. Therefore, *RP11-624C23.1* or *RP11-203E8* silencing can result in increasing resistance to genotoxic stress. Since the genotoxic agents are essential parts of chALL treatment, these lncRNAs may be novel prognostic biomarkers and treatment targets in ALL (117).

### Epigenetic-Based Biomarkers

Epigenetic modifications may have an impact on gene expression, and play essential role in the emergence and recurrence of cancer, therefore may be considered as cancer prognostic biomarkers with important clinical implications (18, 59, 122). In order to identify epigenetic alterations, genome-wide methylation profiles were created for example, using methylated CpG island recovery assays followed by NGS (118), through which, technical standardizations are performed to determine useful clinical disease thresholds for the specific DNA methylation biomarkers (18).

Aberrant DNA methylation patterns are identified to occur during the progression of leukemia and correlated with clinical outcome. Methylation profile analysis of some genes including *CDH1, p73, p16, p15, p57, NES-1, DKK-3, CDH13, p14, TMS1, APAF-1, DAPK, PARKIN, LATS1*, and *PTEN* demonstrated that children including 88 B-ALLs and 12 T-ALLs, with methylation of 4 or more of these genes had worse DFS and OS. Interestingly, promoter methylation of these genes was correlated with favorable outcome in children with t(9;22)(q34;q11) translocation or high white blood cell (WBC) count at diagnosis (122). In addition, it has been demonstrated that hyper-methylation of *p15* is related to worse 8-year DFS in chALL (54 B-ALL and 39 T-ALL) (123).

Methylation-dependent silencing of *p21* was observed in 109 children with ALL (the percentage of B- and T-ALL is not unidentified in this study). *p21* expression is induced by functional p53 protein following apoptosis or cell cycle arrest. Since *p21* methylation is correlated with unfavorable 7-year DFS and 9-year OS, hyper-methylation of *p21* is suggested as an important factor in predicting the clinical outcome in chALL (124).

Methylation analysis of calcitonin (*CALC1*) gene identified the association of *CALC1* hyper-methylation with a higher relapse and mortality rate in chALL (including 56 chALL with unidentified contribution of B- and T-ALL). Downregulation of *p57KIP2* was also shown in patients with *CALC1* hyper-methylation. However, hyper-methylation of *CALC1* gene is associated with increased risk of relapse and worse clinical outcome in chALL (125). Furthermore, downregulation of human large tumor suppressor 2 (*LATS2/KPM*) gene following hyper-methylation is found in chALL with increased risk of relapse and mortality (54 B and T-ALL cases) (126).

In one study, a genome-wide analysis of promoter associated CpG island methylation was performed by the use of both methylated CpG island amplification (MCA) and representational differential analysis (RDA) or a DNA promoter microarray in patients with ALL. Methylation of multiple CpG islands, including *GIPC2, MAGI1, RSPO1, ADCY5, CAST1, OCLN, HSPA4L, MSX2, EFNA5, GNA14, GFPT2, SALL1, ZNF382, MYO5B*, and *MN1* genes, was correlated with worse OS. Patients with more than four methylated genes had a remarkably poorer outcome when treated with hyper CVAD (cyclophosphamide, vincristine, doxorubicin, adriamycin, and dexamethasone) based chemotherapy, used for ALL treatment (127).

It has been identified that Polo-like kinase 2 (*PLK2*) methylation-dependent transcriptional silencing occurs in B cell lymphomas, 100% of Burkitt lymphoma (BL) cell lines and a similarly high proportion of primary BL. The *PLK2* has been lately introduced as a potential prognostic biomarker in response to the chemotherapy. *PLK1* can act as an oncogene and indeed is a prominent target for developmental therapeutics in oncology, while *PLK2*-*PLK5* have properties more similar to tumor suppressor genes (128).

In one study, analysis of methylation-dependent transcription regulation identified a subset of hyper-methylated and down-regulated genes in BCP-ALL, including *CDKN2A, CSMD1, COL6A2*, and *PTPRO*, whereas other genes were hypo-methylated and overexpressed including *NOTCH4* and *TOP1MT* in relapsed children with BCP-ALL (56).

Aberrant methylation of *IRF8, TAL1, MEIS1, IRF4, HOXA4*, and *HOXA5* has been observed in BCP-ALL patients. *MEIS1* and *HOXA4* expression and *TAL1* methylation are correlated with WBC count, National Cancer Institute (NCI) risk classification and age, respectively. *MEIS1* expression is inversely associated with WBC count, *HOXA4* expression is down-regulated in patients with high risk based on the NCI classification and *TAL1* methylation is rather increased in patients older than 9 years and in patients who show relapse (129). It has also been demonstrated that increased histone H4 acetylation is significantly associated with favorable RFS, EFS, OS in pediatric BCP-ALL (130). The identified epigenetic-based biomarkers are introduced in Table 3.

**Table 3:** Epigenetic- and protein-based prognostic biomarkers in pediatric ALL.

**Biomarker**	**Type of biomarker/abnormality**	**Favorable(F)/** **Unfavorable (U) prognosis**	**Lineage**	**References**
**Epigenetic-based**				
*CDH1, p73, p16, p15, p57, NES-1, DKK-3, CDH13, p14, TMS1, APAF-1, PARKIN, LATS1, PTEN*	Hyper-methylation	U	B, T	(122)
*P15*	Hyper-methylation	U	B, T	(123)
*p21*	Hyper-methylation	U	B, T	(124)
*CALC1*	Hyper-methylation	U	B, T	(125)
*LATS2/KPM*	Hyper-methylation	U	B, T	(126)
*GIPC2, RSPO1, MAGI1, CAST1, ADCY5, HSPA4L, OCLN, EFNA5, MSX2, GFPT2, GNA14, SALL1, MYO5B, ZNF382, MN1*	Hyper-methylation	U		(127)
*PLK2*	Hyper-methylation	U	Burkitt	(128)
*CDKN2A, CSMD1, COL6A2, PTPRO*	Hyper-methylation	U	B	(56)
*NOTCH4, TOP1MT*	Hypo-methylation	U	B	(56)
*HOXA4, TAL1, MEIS1*	Hyper-methylation	U	B	(129)
Histone H4	Hyper-acetylation	F	B	(130)
**Protein-based**				
PLK1	Phosphorylation	U	B, T	(131)
ABCA2, ABCA3	Upregulation	U	B, T	(132)
Smo	Upregulation	U	B	(133)
CLUS, CERU, FCN3, APOA1, APOE, APOA4, ACTB, CATA, AFAM, AMBP	Downregulation	U	B	(60)
GELS, S10A9	Upregulation	U	B	(60)
P16^ink4a^	Downregulation	F	B, T	(134)
PTEN (S380), PARP cleaved, caspase 7 cleaved, PDK (S241), PKAc (T197), p90RSK (S380), MEK 1/2 (S217-221), IKBA (S32), GRB2, beta catenin	Upregulation and downregulation	F	B	(135)
YY1, MDR1	Upregulation	U	B	(88)

*CDH1, Cadherin 1; NES-1, Normal epithelial cell-specific 1; DKK-3, Dickkopf WNT signaling pathway inhibitor 3; CDH13, Cadherin 13, TMS1, Target of methylation induced silencing; DAPK, Death-associated protein kinase; LATS1, Large tumor suppressor kinase 1; PTEN; Phosphatase and tensin homolog; CALC1, Calcitonin; LATS2, Large tumor suppressor 2; GIPC2, GIPC PDZ domain containing family member 2; RSPO1, R-spondin 1; MAGI1, Membrane associated guanylate kinase, WW and PDZ domain-containing protein 1; CAST1, Cytosolic arginine sensor for MTORC1 subunit 1; ADCY5, Adenylate cyclase 5; HSPA4L, Heat shock protein family A (Hsp70) member 4 Like; OCLN, Occludin; EFNA5, Ephrin A5; MSX2, Msh homeobox 2; GFPT2, Glutamine-fructose-6-phosphate transaminase 2; GNA14, G protein subunit alpha 14; SALL1, Spalt like transcription factor 1; MYO5B, Myosin VB; ZNF382, Zinc finger protein 382; MN1, Meningioma 1; PLK2, Polo-like kinase 2; CDKN2A, Cyclin-dependent kinase Inhibitor 2A; PTPRO, Protein tyrosine phosphatase receptor-type O; NOTCH4, Notch receptor 4; TOP1MT, Topoisomerase (DNA) I, mitochondrial; HOXA4, Homeobox A4; TAL1, TAL BHLH transcription factor 1; MEIS1, Meis homeobox 1; PLK1, Polo-like kinase 1; ABCA2, ATP binding cassette subfamily A member 2; ABCA3, ATP binding cassette subfamily A member 3; CLUS, Clusterin; CERU, Ceruloplasmin; FCN3, Ficolin 3; APOA1, Apolipoprotein A1; APOE, Apolipoprotein E; APOA4, Apolipoprotein A4; ACTB, Actin beta; CATA, Catalase; AFAM, Afamin; AMBP, Alpha-1-microglobulin/bikunin precursor; GELS, Gelsolin; S10A9, S100 calcium binding protein A9 gene; PARP, Poly [ADP-ribose] polymerase 1; PDK, Pyruvate dehydrogenase kinase; PKAc, cAMP-dependent protein kinase catalytic subunit; p90RSK, 90 kDa ribosomal s6 kinase; IKBA, Ikappabalpha; GRB2, Growth factor receptor bound protein 2; YY1, Yin Yang 1; MDR1, Multi-drug resistance-1*.

### Protein-Based Biomarkers

The gene expression alterations induced at protein levels may be attributed to post-transcriptional, translational and post-translational modifications (8). The applied post-translational modifications, such as glycosylation or phosphorylation, in addition to the protein up or down regulation, may be considered as protein-based biomarkers in various diseases (7). These alterations may be identified using immunoassay formats, electrophretic techniques and targeted mass spectrometry. The protein-based biomarkers recently introduced in ALL are shown in Table 3.

It has been newly shown that PLK1 protein phosphorylation on Thr210 is prevalent in chALL (including 168 B-ALL and 34 T-ALL) and associated with lower EFS. PLK1 phosphorylation contributes to its binding to p53 and inhibiting its pro-apoptotic function. while, in turn, PLK1 expression is transcriptionally inhibited by p53 upon DNA damage, showing a role for PLK1 in the G2/M phase of proliferation (131).

Based on our previous findings, the overexpression of ABCA2 and ABCA3 transporters at protein levels can have prognostic impact on chALL (60 B-ALL and 9 T-ALL). Tertiary structure and docking analyses showed a possible compensatory mechanism between these two ABC transporters, through which, up-regulation of one of them may lead to drug efflux and multidrug resistance (132). Furthermore, our group has recently identified that overexpression of Smo at the protein level following the downregulation of *miR-326* is related to MDR in pediatric B-ALL. Our results revealed a positive correlation between Smo and ABCA3 transcripts in these children (133).

Braoudaki et al. suggested that CLUS, CERU, FCN3, GELS, APOA1, APOE, APOA4, S10A9, ACTB, CATA, AFAM, and AMBP proteins can be considered as potential prognostic markers for aggressive pediatric B-ALL. In this study, aggressive B-ALL patients with high risk of relapse, were those with aged <1 or >10 years old, WBC count > 50 × 10^9^/L, L2 > 20% or L3 blasts and CNS disease at diagnosis. In addition, down-regulation of GELS and S10A9 proteins were introduced as poor prognostic biomarkers in these children. Up-regulation of FCN3, ApoE, ApoA1, ApoA4, AMBP, ACTB, CATA, AFAM, CLUS, and CERU proteins has also been observed in aggressive B-ALL patients (60).

p16^ink4a^ is a tumor suppressor protein and cyclin-dependent kinase inhibitor (CKI), which results in G1 arrest in the presence of a functional pRb. High expression of this protein was shown in chALL with inferior DFS, whereas p16^ink4a^ low expression was a favorable prognostic factor. Therefore, it has been suggested that p16^ink4a^ expression at protein level is a strong prognostic biomarker in chALL. Forty three B-ALL and 15 T-ALL were investigated in this study (134).

One study demonstrated that a subgroup of pediatric B-ALL patients with specific pattern of protein expression including higher level of PTEN (S380), cleaved PARP, cleaved caspase 7, PDK (S241), PKAc (T197), p90RSK (S380), MEK 1/2 (S217-221), IKBA (S32), and GRB2 and lower beta catenin expression have superior survival (135).

Recently, immunocytochemistry analysis revealed that overexpression of YY1 at the protein level is correlated with unfavorable OS via upregulation of MDR1/P-gp in chALL (including 51 B-ALL and 12 T-ALL). Overexpression of MDR1 protein contributes to the resistance to a variety of chemotherapeutic drugs and relapse in chALL (88).

## ALL Predictive Biomarkers

Predictive biomarkers give information about the response to a specific treatment and help to select particular treatment over another (Table 4), while prognostic biomarkers provide information about the natural history of the disorder (145). In other words, predictive biomarkers can be used to recognize individuals who are more likely to respond to a particular medical reagent or environmental agent. The aforementioned response can be a symptomatic benefit, better survival, or a detrimental effect (146). More precisely, the predictive biomarkers help to optimize therapy decisions and facilitate personalized chemotherapy (147).

**Table 4:** Predictive biomarkers in pediatric ALL.

**Predictive biomarker**	**Type of biomarker**	**Type of abnormality**	**Favorable(F)/** **Unfavorable (U)**	**Drug therapy** **-Type of chALL**	**Lineage**	**References**
*MLL*-rearrangements	DNA	Translocation	U	Glucocorticoids	B	(136)
*MLL-AF4*	DNA	Translocation	U	Glucocorticoids	B	(137)
*CD200*/*BTLA*	DNA	Deletion	U	EORTC-CLG 58951 protocol	B	(138)
*MSH6*	DNA	Deletion	U	Thiopurines and alkylators	B	(56)
*BTG1, NR3C1*	DNA	Deletion	U	Glucocorticoids	B	(56)
*NT5C2*	DNA	Mutation	U	6-mercaptopurine – B-ALL and T-ALL	B, T	(139)
*SLCO1B1, ABCC2, ABCC4*	DNA	Polymorphism	U	Methotrexate	B	(140)
*miR-18a, miR-218, miR-532*	miRNA	Upregulation	F	Prednisone	B, T	(107)
*miR-193a, miR-625, miR550, miR-638*	miRNA	Downregulation	F	Prednisone	B, T	(107)
*miR-128b, miR-221*	miRNA	Downregulation	U	Prednisone	*MLL-AF4*	(137)
*miR-454*	miRNA	Downregulation	U	L-asparaginase	B, T	(103)
*miR-125b, miR-99a, miR-100*	miRNA	Upregulation	U	Vincristine and daunorubicin	B, T	(103)
*miR-17*	miRNA	Upregulation	U	Dexamethasone	B	(141)
*BALR-2*	LncRNA	Upregulation	U	Glucocorticoids	*MLL*-rearranged	(142)
*AKR1C3*	mRNA	Upregulation	F	PR-104	T	(143)
*BIM*	mRNA and protein	Downregulation	U	Dexamethasone	B	(141)
*BIM*	mRNA and protein	Upregulation	F	Prednisolone	B	(144)
*PCNA*	mRNA and protein	Downregulation	F	Prednisolone	B	(144)
*VDAC1*	mRNA and protein	Upregulation	U	Prednisolone	B	(70)

*CD200/BTLA, CD200 molecule/B and T lymphocyte associated; MSH6, MutS homolog 6; BTG1, BTG anti-proliferation factor 1; NR3C1, Nuclear receptor subfamily 3 group C member 1; NT5C2, 5′-nucleotidase, cytosolic II; SLCO1B1, Solute carrier organic anion transporter family member 1B1; ABCC2:ATP binding cassette subfamily C member 2; ABCC4, ATP binding cassette subfamily C member 4; AKR1C3, Aldo-keto reductase family 1 member C3; PCNA, Proliferating cell nuclear antigen; VDAC1, Voltage dependent anion channel 1; cTnT, Cardiac Troponin; NT-proBNP, N-terminal pro b-type natriuretic peptide*.

As discussed above, *MLL* translocation is correlated with poor prognosis in chALL. In one study, it is revealed that patients with *MLL* rearrangement, specially infants, show GC resistance (136), introducing this translocation as a predictive biomarker.

*CD200*/*BTLA* deletions act as predictive biomarkers in pediatric BCP-ALL without any known genetic lesions, and are associated with <8-year EFS. These patients are treated according to the EORTC-CLG 58951 protocol. A strong correlation has been observed between *CD200*/*BTLA* deletions and *ETV6*-*RUNX1* positive leukemia as well. *ETV6*-*RUNX1* positive is a good prognostic biomarker in *BCP-ALL* and *CD200*/*BTLA* deletions do not affect prognosis within this genetic subtype (138). It has been found that specific deletions of *MSH6, BTG1*, and *NR3C1* have been related to resistance to thiopurines, alkylators (*MSH6*) and GCs (*BTG1* and *NR3C1*) in pediatric B-ALL (56). Furthermore, mutations in the cytosolic 5′-nucleotidase II gene *NT5C2* lead to proteins with increased nucleotides activity and result in increased nucleoside-analog metabolism and 6-mercaptopurine resistance in chALL (103 T-ALL and 35 BCP-ALL) (139).

Moreover, it has been shown that *SLCO1B1* rs11045879, *ABCC2*rs3740065, and *ABCC4*rs9516519 polymorphisms are novel predictive biomarkers for Methotrexate (MTX) toxicity and outcome in pediatric B-ALL. The association between these polymorphisms and the toxicity of MTX has been confirmed through analysis of 384 SNPs in twelve MTX transporter genes (*SLCO1B1, SLCO1B3, SLCO1A2, ABCG2, ABCB1, ABCC1, ABCC2, ABCC3, ABCC4, SLC19A1, SLC22A6*, and *SLC22A8* (140).

It has been shown that upregulation of *miR-193a, miR-625, miR-550*, and *miR-638* is associated with resistance to prednisone, while *miR-18a, miR-218*, and *miR-532* were downregulated in children with poor prednisone response (The ALL samples in this study consisted of 48 B-ALL and 1 T-ALL) (107). Downregulation of *miR-128b* and *miR-221* are predictive biomarkers for resistance to GC in pediatric *MLL-AF4* ALL cells. *MLL-AF4* translocation is correlated with GC resistance and is introduced as a predictive biomarker in chALL carrying this translocation. Restoration of these miRNAs can sensitize *MLL-AF4* ALL cells to GC drugs (137). Furthermore, low expression of *miR-454* is associated with resistance to L-asparaginase (in 81 patients), while overexpression of *miR-125b, miR-99a*, and *miR-100* is related to vincristine and daunorubicin resistance in chALL (103). It has also been demonstrated that overexpression of miR-17 and downregulation of its target (BIM) result in dexamethasone resistance in childhood B-ALL (141).

LncRNA *BALR-2* can promote cell survival via inhibition of downstream genes of the GC receptors, such as *Fos, Jun*, and *BIM*. *BALR*-2 plays a role in resistance to glucocorticoid-induced apoptosis and up-regulates in *MLL*-rearranged BCP-ALL. *BIM* has an indispensable role in the regulation of B-ALL GC response and can be served as a predictive biomarker in pediatric B-ALL (142).

According to the *in vivo* and *in vitro* studies, AKR1C3 is introduced as a predictive biomarker for response to PR-104 treatment, in pediatric T-ALL. In patient-derived leukemic blasts, primary T-ALL cells have been proven to be more sensitive to PR-104 than BCP-ALL primary cells, and this sensitivity correlates with AKR1C3 expression. It has been suggested that PR-104 represents a new treatment for refractory/relapsed T-ALL, and expression of AKR1C3 can be served as a predictive biological marker to detect those children who will most likely take advantage of therapy with PR-104 in prospective clinical trials (143).

Early response to 7 days-PRED treatment is one of the most prominent positive prognostic factors in pediatric B-ALL. Patients with up regulation of the two proteins named, BIM and voltage-dependent anion-channel protein1 (VDAC1), at day eight of PRED monotherapy, showed increased EFS compared with those whose gene expression profiles were not changed followed by PRED therapy. Down regulation of the proliferating cell nuclear antigen (PCNA) revealed similar impact on patients EFS. These data introduced three promising predictive biomarkers of BMI, VDAC1, and PCNA in childhood B-ALL (70, 144).

## Circulating Biomarkers

Due to the promising characteristics of high stability, low cost, possibility of repeated sampling and noninvasiveness, circulating biomarkers have received extensive attention in molecular medicine nowadays. Compelling evidences have shown that these types of biomarkers are measurable in secreted membrane vesicles (microvesicles and exosomes), blood serum, and other body fluids and can be used as biomarkers to diagnose, classify, follow-up and predict prognosis in chALL (96, 98–101). It has been suggested that extracellular circulating biomarkers play an important role in intracellular communication in both paracrine and endocrine manners (101).

Serum vascular endothelial growth factor-A (VEGF-A) is recently introduced as a circulating biomarker which can be useful for screening high-risk chALL patients and monitoring their response to treatment (148). A study has suggested that low serum levels (≤60 pg/mL) of the VEGF-A, at the end of induction (day 28), is significantly correlated with favorable 6-year EFS in children with standard risk B-ALL. Whereas, children with high VEGF-A serum levels (>100 pg/mL) or a rise in the VEGF-A level during induction (days 0–28) have shown worse 6-year EFS. Furthermore, VEGF-A serum concentration at the entry into induction (day 0) is reported to be related to time of relapse. Another study demonstrated that the mean values of serum Fas at the time of diagnosis is associated with response to treatment in 29 chALL (with unidentified portion of B- and T-ALL). In other words, relapsed children have higher Fas levels compared with patients in CR. Therefore, serum Fas levels are appropriate circulating biomarkers for chALL follow up (149). It has also been demonstrated that higher serum levels of interleukin 2 receptor (IL2R) (>2.000 U/mL) is a useful circulating biomarker for the prediction of response to treatment in children with non-T, non-B ALL, but not in those with T cell disease. However, increased levels of IL2R is related to the worse treatment outcome in these patients (150). Another study revealed that at day 14 and day 29 of induction, significant decreased serum lactate dehydrogenase (LDH) level is associated to increased platelet count and decreased peripheral and bone marrow blast cell percentages in chALL (including 44 patients with unidentified percentage of B- and T-ALL) (151).

To the best our knowledge, only one circulating miRNA has already been introduced to be correlated with response to treatment and outcome in chALL. It has been recently demonstrated that circulating miR-652-3p is downregulated in childhood B-ALL at initial diagnosis and relapse compared with the samples of the same patients at CR. In addition, miR-652-3p upregulation using mimic leads to vincristine- and cytarabine-induced apoptosis in lymphoblast cells via increased sensitivity to these drugs (152) (Table 5).

**Table 5:** Circulating biomarkers in pediatric ALL.

**Circulating biomarker**	**Type of biomarker**	**Type of abnormality**	**Favorable (F)/** **Unfavorable (U)**	**Lineage**	**References**
VEGF-A	Protein	High level	U	B	(148)
Fas	Protein	High level	U	B, T	(149)
IL2R	Protein	High level	U	non-T, non-B	(150)
LDH	Protein	High level	U	B, T	(151)
*miR-652-3p*	miRNA	Downregulation	U	B	(152)

*VEGF-A, Vascular endothelial growth factor-A; IL2R, Interleukin 2 receptor; LDH, Lactate dehydrogenase*.

## Patient-Related Biomarkers

Some clinical and hematological factors play important role in chALL patients response to chemotherapy, including age, WBC and leukocyte count at initial diagnosis, immunophenotype, involvement of CNS, the presence or absence of mediastinal tumor, and mrd in different stages of treatment (37, 153). Children between 1 and 10 years of age have a favorable prognosis and better outcome compared to the children younger than 24 months, adolescents and adults. However, these factors are used to classify chALL patients into the different risk groups including standard risk with 90% survival rate, intermediate risk with 60% rate survival and high risk with 30% survival rate (37). For example, children with WBC count under 50,000/μL, at diagnosis, show better response to treatment than patients with WBC count equal to or above 50,000/μL (154). The leukocytosis count >30 × 10^9^L and >100 × 10^9^L is identified as a poor prognostic factor in pediatric B-ALL and T-ALL, respectively.

Children with T-ALL and pro B-ALL immunophenotypes as well as mrd-positive children have worse outcome and EFS (155, 156). It has been demonstrated that pediatric B-ALL patients with CD10^−^ or CD34^−^ have poor outcome compared with those who are CD10^+^ or CD34^+^ (157).

## Therapy-Related Biomarkers

One of the most important limitations in chALL treatment is acquired resistance to treatment, which is developed after exposure to chemotherapy agents and contributed to MDR and relapse (158). Identification of the biomarkers related to drug resistance may provide novel approaches to targeted therapy and improve patient clinical outcome. Therapy-related biomarkers are shortly introduced in this section.

Deletions/Mutations of the tumor suppressor gene *TP53* are correlated with poor response to treatment, EFS, and OS in chALL (218 BCP-ALL and 47 T-ALL). Alterations in *TP53* gene were shown to be gained in relapsed patients after treatment, using direct sequencing and multiplex ligation-dependent probe amplification in 23 matched samples (159).

DNA analysis of paired chALL samples at diagnosis, relapse, or complete remission (CR) revealed that some missense mutations specially occur in relapsed patients after treatment and are not identified at initial diagnosis or CR. These mutations happen in *RGS12, LPHN1, CAND1, PRMT2, NIPSNAP1, USP7, TULP4, CBX3, COBRA1, SDF2, FBXO3, SCARF1, NEGR1, NT5C2, DPH5, SMEK2, MIER3, DOPEY1, ZNF192*, and *NT5C2* genes. RNA sequencing of 10 matched cases demonstrated that novel somatic mutations are acquired in *NT5C2* gene at relapse and are not presented at diagnosis. Enzymatic analysis in additional 61 relapsed cases identified that these mutations conferred increased enzymatic activity of the protein cN-II, a 5′-nucleotidase, and resistance to nucleoside analog therapies (160).

MicroRNA expression analysis of paired diagnosis/relapse samples (11 B-ALL and 6 T-ALL patients) revealed that some miRNAs are differentially expressed at diverse stages of the disease. *miR-223, miR-23a*, and *miR27a*, are examples of those miRNAs which are downregulated at initial diagnosis, restored at CR, and downregulated once again at relapse. On the other hand, some miRNAs, including *miR-181a, miR-181b, miR-128b*, and *miR-708* are overexpressed at both diagnosis and relapse. These sets of miRNAs may have main roles in leukemogenesis and post-therapy drug resistance (161).

Methylation analysis of some genes in 8 patients with B-ALL and 1 with T-ALL showed hyper methylation of *MGMT*, and *p16* at both diagnostic and relapse status. However, *RARB* was specifically hyper-methylated at the time of relapse. It is suggested that, *RARB* hyper-methylation is correlated with chALL progression and should be monitored after chemotherapy (162).

High-throughput analysis of matched diagnosis/relapse childhood BCP-ALL samples revealed more frequent somatic deletions in *KZF1, VPREB1, NR3C1*, and *EBF1* genes at the stage of relapse compared with the disease onset (56). Furthermore, in contrast to T-ALL (163), BCP-ALL showed significantly more hyper-methylated genome at relapse (56). Promotor hyper-methylation, and consequently downregulation of *CDKN2A, PTPRO*, and *CSMD1* tumor suppressor genes was also identified only in relapsed chALLs, but not those at the onset of the disease. Furthermore, some genes including *FANCD2, FOXM1, CENPM*, and *OBSL1* were only expressed at relapse, not diagnostic status of pediatric BCP-ALL (56).

One study has identified that, increased serum levels of cTnT (cardiac injury biomarker) and N-terminal pro-brain natriuretic peptide (NT-proBNP; cardiomyopathy biomarker), followed by treatment with doxorubicin, may act as biomarkers for cardiac injury and cardiomyopathy in children with high-risk ALL (52 T-ALL and 434 B-ALL) (164). Although Doxorubicin chemotherapy is very effective in pediatric ALL, it may also cause damage to myocardial cells and consequently, induce cardiomyopathy (164, 165) (Table 6).

**Table 6:** Therapy-related biomarkers in pediatric ALL.

**Therapy-related biomarker**	**Type of biomarker**	**Type of abnormality**	**Favorable(F)/** **Unfavorable (U)**	**Lineage**	**References**
*TP53*	DNA	Mutation/deletion	U	B, T	(159)
*NT5C2*	DNA	Mutation	U		(160)
*miR-223, miR-23a, miR27a*	miRNA	Downregulation	U	B, T	(161)
*miR-181a, miR-181b, miR-128b, miR-708, miR-130b*	miRNA	Upregulation	U	B, T	(161)
*RARB*	Epigenetic	Hyper-methylation	U	B, T	(162)
*CDKN2A, PTPRO, CSMD1*,	Epigenetic	Hyper-methylation	U	B	(56)
*CDKN2A, PTPRO, CSMD1, FANCD2, FOXM1, CENPM, OBSL1*	mRNA	Downregulation	U	B	(56)
*cTnT, NT*-*proBNP*	Protein	Upregulation	U	B, T	(164)

*TP53, Tumor protein P53; NT5C2, 5′-nucleotidase, cytosolic II; CDKN2A, Cyclin-dependent kinase Inhibitor 2A; PTPRO, Protein tyrosine phosphatase receptor-type O; CSMD1, CUB and sushi multiple domains 1; FANCD2, FA complementation group D2; FOXM1, Forkhead box M1; CENPM, Centromere protein M; OBSL1, Obscurin-like protein 1; cTnT, Cardiac troponin; NT-proBNP, N-terminal pro b-type natriuretic peptide*.

## Biomarkers in Personalized Medicine and Immunotherapy

Since the effectiveness and cure rate of conventional chemotherapy depend on many individual factors, such as genetic and protein expression differences in one patient vs. another, the field of precision and personalized medicine is growing incredibly fast (166). Some of the diagnostic, prognostic, and predictive biomarkers are currently used in clinical practice of personalized and targeted oncotherapy, such as: tyrosine kinase inhibitors, e.g., Gleevec® (imatinib; Novartis) in ALL patients with *BCR-ABL1* gene rearrangements; FLT3 inhibitor Hydrate® (CEP-701, lestaurtinib; Cephalon Inc.) in ALL patients with *MLL* rearrangements; and purine nucleoside phosphorylase inhibitor Mundesine® (forodesine; Mundipharma AG) in pediatric T-ALL with purine nucleoside phosphorylase mutation (167, 168).

Immunotherapy is another more specific and effective class of cancer treatments. Some of the pediatric immune-based approaches have received FDA approval, including monoclonal antibodies (mAbs), checkpoint inhibitors, bispecific T-cell engagers (BiTEs), and chimeric antigen receptor-modified T (CAR-T) cells. However, some immunotherapy protocols are still under way to be examined, such as vaccines and oncolytic virotherapies (169). CAR-T cells are genetically engineered to target surface antigens on tumor cells and have exhibited good efficacy for treatment of the hematological malignancies (170). For example, tisagenlecleucel (also known as Kymriah or CTL019) is a CD19 CAR-T cell, which is used for immunotherapy in pediatric B-ALL (169). There are evidences indicating post CR-relapse followed by CD-19 CAR-T infusion. In these cases, transfusion of CD22 and/or CD20 CAR-T cells followed by CD-19 CAR-T cell therapy may decrease the side effects raised by the first round immunotherapy. Although much has been explored to this regard, CAR-T cell therapy requires closer inspection and there is a growing need to determine biomarkers in order to improve response to immunotherapy (171).

## Challenges in Clinical Applications of Biomarkers

Due to the important role of biomarkers at all stages of disease, it is essential that they undergo extremely thorough and careful evaluations, such as analytical and clinical validations and clinical utility assessments, before incorporation into a routine clinical care (1). Identification, validation and introduction of a novel biomarker is just as difficult as the development and approval of a new drug. Moreover, ~30–50% of biomarkers are coupled to drug development programs and only 3–5% reach the clinical usage (172). Herein, we point out some challenges in the validation of biomarkers and their application in chALL clinical trials.

A number of biological challenges are encountered during the development of novel biomarkers, including the intrinsic biological differences among distinct individuals, complexity in tumor response to treatment and the complexity of various biological systems, involving diverse interacting molecular pathways and adaptive feedback and cross-talk loops. Besides the aforementioned challenges, various types of measurement errors may occur during the validation of disease biomarkers (4, 173, 174). The fundamental determinant assuring the reliability of biomarkers analyses is the use of standard protocols for sample collection, processing, and storage (172). On the other hand, the statistical analyses of the biomarker data is another main logistical component of the validation process; statistical evaluation is challenging regarding achievement of uniformity in data management, biostatistics, and bioinformatics methodologies (172). In biomarker validation, statistical approach must detect relationships that happen by chance from those reflecting true biological associations (175).

However, omic features (i.e., genomics, proteomics, epigenetics, etc.) and/or environmental and lifestyle factors, may contribute to drug response. It is necessary to recognize biomarkers through either omic data alone or along with environmental/lifestyle factors such as age, gender, diet, and other metabolic factors, studied in personalized medicine, to use the “right drug,” for the “right patient,” at the “right dose,” at the “right time” (4, 176).

In order to identify valid and reliable biomarkers, cohort studies should be performed, evaluating large number of sample collections (177). In this regard, one of the main challenges to identify new biomarkers in chALL is the limited number of children participating in research studies because of the painful or invasive procedures required for sample collection. Another challenge is obtaining age-matching control samples, which is essential to identify normal reference value. In addition to the low number of childhood malignancies compared with adults, parents are reluctant to participate in the trials (178).

## Conclusion

In conclusion, MDR is a serious impediment to the treatment of chALL; the most common malignancy in children. Treatment sensitivity/drug resistance depends on many factors, such as genetic, epigenetic, transcriptomic, and proteomic alterations. These modifications can be used as bio-signatures, helping better risk stratification and, predicting patient response to therapy. Some chromosomal translocations are introduced as positive prognostic biomarkers, including t(1;19)(q23;p13), t(12;21)(p13;q22), t(7;10)(q35;q24), and t(10;14)(q24;q11.2), which may predict a favorable outcome; Some others are presented as negative bio-forecasters including t(17;19)(q22;p13), t(9;22)(q34;q11), t(8;14)(q24;q32), and t(7;19)(q35; p13), which anticipate worse response to treatment. Some of the drug resistance biomarkers are currently used for targeted therapy. Examples of these molecules are BCR-ABL1 gene rearrangement which is targeted by Imatinib, the commercially fabricated tyrosine kinase inhibitor, and MLL rearrangement which is potentially hampered by the FLT3 inhibitor, Lestaurtinib. Moreover, pediatric chALL biomarkers may be attacked using immune-based approaches including CAR-T cell therapies. Circulating biomarkers, are promising biological markers which are detected non-invasively, and employed for classification, follow-up and predicting prognosis in chALL.

## Author Contributions

NA and EG conceived the ideas and wrote the article. KG reviewed the article and made suggestions. SR conceived the ideas, wrote and reviewed the article, and made suggestions.

### Conflict of Interest

The authors declare that the research was conducted in the absence of any commercial or financial relationships that could be construed as a potential conflict of interest.
